# A Urodynamic Comparison of Neural Targets for Transcutaneous Electrical Stimulation to Acutely Suppress Detrusor Contractions Following Spinal Cord Injury

**DOI:** 10.3389/fnins.2019.01360

**Published:** 2019-12-17

**Authors:** Sean Doherty, Anne Vanhoestenberghe, Lynsey Duffell, Rizwan Hamid, Sarah Knight

**Affiliations:** ^1^Aspire Centre for Rehabilitation Engineering and Assistive Technologies, University College London, London, United Kingdom; ^2^London Spinal Cord Injury Centre, Royal National Orthopaedic Hospital, London, United Kingdom

**Keywords:** neuromodulation, spinal cord injury, bladder, incontinence, neurogenic detrusor overactivity, electrical stimulation

## Abstract

**Objectives:**

To assess and compare the effect of transcutaneous Dorsal Genital Nerve Stimulation (DGNS), Tibial Nerve Stimulation (TNS), Sacral Nerve Stimulation (SNS), and Spinal Stimulation (SS) on Neurogenic Detrusor Overactivity (NDO) and bladder capacity in people with Spinal Cord Injuries (SCI).

**Materials and Methods:**

Seven male participants with supra-sacral SCI were tested. Standard cystometry (CMG) was performed to assess bladder activity at baseline and with stimulation applied at each site. This was conducted over four separate sessions. All stimulation was monophasic, 15 Hz, 200 μS pulses and applied at maximum tolerable amplitude. Results were analysed against individual control results from within the same session.

**Results:**

Dorsal Genital Nerve Stimulation increased bladder capacity by 153 ± 146 ml (*p* = 0.016) or 117 ± 201%. DGNS, TNS and SNS all increased the volume held following the first reflex contraction, by 161 ± 175, 46 ± 62, and 34 ± 33 ml (*p* = 0.016, *p* = 0.031, *p* = 0.016), respectively. SS results showed small reduction of 33 ± 26 ml (*p* = 0.063) from baseline bladder capacity in five participants. Maximum Detrusor Pressure before leakage was increased during TNS, by 10 ± 13 cmH_2_O (*p* = 0.031) but was unchanged during stimulation of other sites. DGNS only was able to suppress at least one detrusor contraction in five participants and reduced first peak detrusor pressure below 40 cmH_2_O in these 5. Continuous TNS, SNS, and SS produced non-significant changes in bladder capacity from baseline, comparable to conditional stimulation. Increase in bladder capacity correlated with stimulation amplitude for DGNS but not TNS, SNS or SS.

**Conclusion:**

In this pilot study DGNS acutely suppressed detrusor contractions and increased bladder capacity whereas TNS, SNS, and SS did not. This is the first within individual comparison of surface stimulation sites for management of NDO in SCI individuals.

## Introduction

Neural control of the bladder during storage and micturition involves complex interactions between centres in the brain, spinal cord and peripheral nerves. Intact somato-visceral sacral reflexes are essential for natural voiding. The aberration of pelvic functions following Spinal Cord Injury (SCI) involves the development of uncontrolled and overactive reflex arcs, leading to Neurogenic Detrusor Overactivity (NDO) and Detrusor Sphincter Dyssynergia (DSD) (Craggs et al., 2006). NDO leads to high intravesical pressures, with potential harm done to the upper urinary tract, and urinary incontinence. DSD and inability to produce a sustained detrusor contraction inhibit the ability to void.

Improvements have been made in treatment options available for managing NDO following SCI. The range of antimuscarinic medication (AM), introduction of β3-agonists (Wöllner and Pannek, 2016) and success of Botulinum-A toxin injections (BTX) (Reitz et al., 2004) contribute to satisfactory management of the bladder post SCI. However, some patients remain refractory to current treatment, indeed 56% of 142 United Kingdom patients surveyed experienced at least monthly incontinence episodes, with significant correlation between incontinence episodes and lower quality-of-life scores (Liu et al., 2010). Additionally, the long-term effects of AM remain unclear (Gray et al., 2015). For refractory cases the major surgical option is cystoplasty. Although this has very good long term results, it is a major undertaking with significant morbidity (Hoen et al., 2017).

Neuromodulation, described as “activity in one neural pathway modulating the pre-existing activity in another through synaptic interaction” (Craggs and McFarlane, 1999), may be used for control of the bladder following SCI in various modes of application. The array of neural interactions involving descending control and spinal reflexes presents several potential targets for external manipulation. Stimulation of the Dorsal Genital Nerve (DGNS), Tibial Nerve (TNS), the Sacral Nerves of S2-4 (SNS) and of the Spinal Cord at the T12 vertebral level (SS) all have evidence suggesting efficacy at improving neurogenic Lower Urinary Tract (LUT) storage dysfunction.

Electrical stimulation of pudendal afferents has been investigated in some detail in humans with SCI, where stimulation of the dorsum of the penis or clitoris (DGNS) can reliably suppress detrusor contractions, reduce incontinence and increase bladder capacity (Nakamura and Sakurai, 1984; Vodusek et al., 1986; Kirkham et al., 2001; Bourbeau et al., 2018a). Stimulated pudendal afferents are projected onto sympathetic and parasympathetic pathways, causing inhibition of the detrusor muscle alongside excitation of the sphincters, a coordinated storage response (Lindström et al., 1983; Reitz et al., 2003). However, the use of DGNS on an ongoing basis presents problems in application and acceptability that could be avoided should stimulation of less intimate sites elicit a comparable suppressive effect on detrusor activity.

Sacral Nerve Stimulation, particularly of the S3 spinal nerve, is a widely used approach for treatment of overactive bladder syndrome and various other non-neurogenic LUT symptoms. It has been used in limited neurogenic cases with mixed results (Wöllner et al., 2016). There have been several reports of improvement in LUT dysfunction using transcutaneous stimulation (Walsh et al., 2001; Barroso et al., 2013; Quintiliano et al., 2015) in addition to implantable devices, as well as a study reporting no effect on NDO in a small MS cohort. An acute inhibitory effect of SNS on NDO has been shown in several animal studies (Snellings and Grill, 2012; Su et al., 2012; Ren et al., 2016). Using non-invasive magnetic stimulation techniques, NDO has been acutely suppressed in humans with SCI (Sheriff et al., 1996), and interestingly, early application of implanted, continuous, SNS in the acute stages of SCI has prevented the development of NDO in the chronic phase of injury (Sievert et al., 2010).

Tibial Nerve Stimulation has primarily been applied percutaneously and intermittently, in 12 weekly sessions of 30 min (Peters et al., 2013). Since the effects of TNS on bladder overactivity were documented by McGuire (McGuire et al., 1983), TNS has also been studied in neurogenic patients with Multiple Sclerosis (MS), Parkinsons (PD) and SCI using a variety of stimulation protocols and outcome measures. When studied acutely, it has been observed to increase bladder capacity (Amarenco et al., 2003; Andrews and Reynard, 2003; Kabay et al., 2008, 2009) and, when used daily, to improve symptoms in MS and PD cohorts, recorded using bladder diaries (de Sèze et al., 2011; Kabay et al., 2017). However, no conclusive evidence has been provided to show the effectiveness or mechanism of TNS (Schneider et al., 2015) and negative results have also been reported in an MS cohort (Fjorback et al., 2007b). There is supporting evidence from animal studies of an acute inhibitory effect on detrusor overactivity (Kovacevic and Yoo, 2015; Gad et al., 2016). Studies have used both trans- and percutaneous stimulation techniques with positive effect.

Spinal Stimulation, using stimulation of the dorsal surface or dorsal roots of the spinal cord at the level of the T12 vertebra has had promising reports of improved lower limb and bladder control from both pre-clinical and clinical studies (Harkema et al., 2011; Hofstoetter et al., 2014; Gerasimenko et al., 2015; Gad et al., 2016; Ren et al., 2016; Herrity et al., 2018). Previous studies of epidural stimulation conducted with MS participants have reported acute improvements in LUT function (Meglio et al., 1980), including in the suppression of detrusor contractions with some carry-over effect noted. Animal work has reported acute suppression of detrusor contractions using both dorsal root and sacral nerve stimulation in rats, whilst stimulation of ventral roots was unable to suppress detrusor activity (Ren et al., 2016). More recently, application of epidural, magnetic and transcutaneous SS have yielded promising results for both storage and voiding, each in pilot studies involving humans with chronic SCI (Gad et al., 2018; Herrity et al., 2018; Niu et al., 2018). It is possible to stimulate these structures using transcutaneous stimulation techniques (Hofstoetter et al., 2018).

Direct comparison of neuromodulation sites has been evaluated in rats (SNS, DGNS, and TNS), cats (DGNS, pudendal trunk, and SNS) (Snellings and Grill, 2012) and in human subjects with MS (DGNS, SNS, and TNS) (Fjorback et al., 2007a, b). It has not been reported previously in humans with SCI.

The purpose of this study was to compare the acute urodynamic effect of transcutaneous stimulation at four anatomical sites, to assess their ability to lower detrusor pressure, reduce incontinence, and increase bladder capacity. This study was performed in participants with chronic SCI and NDO, enabling within-individual comparison of results.

## Materials and Methods

### Approval

This study was carried out in accordance with Good Clinical Practice and NHS guidelines. The protocol was approved by the Queen Square Regional Ethics Committee in the United Kingdom. All subjects gave written informed consent in accordance with the Declaration of Helsinki. The study was retrospectively listed on a clinical trials database, ISRCTN99373118.

### Participants

Ten male subjects with complete or incomplete SCI and history of NDO were recruited into this study. Two were withdrawn during baseline screening as no NDO was found on the day of study and a further one participant withdrew due to unavailability. Other exclusion criteria included BTX in the preceding 6 months or surgery to the LUT. Seven participants completed the study, two of whom were excluded from SS due to metal implants under the stimulation site. Participants stopped taking AM for the 5 days preceding each experimental session. Table 1 outlines the participant characteristics.

**TABLE 1:** Participant characteristics with respect to injury level, ASIA grade, cause of injury (T, trauma, Non-T, not trauma) and age.

**ID**	**Age**	**Sex**	**Injury**	**ASIA**	**Years since injury**	**Cause of injury**	**Bladder voiding**	**Bladder storage**	**Previous BTX**	**Sensation**	**Incontinence**
P01	44	M	T4	A	5	T	ISC + sheath	AM	Y	N	Y
P03	68	M	C5	D	10	Non-T	ISC	AM	Y	Y	Y
P04	50	M	L1	A	1	T	ISC	AM	N	Y	Y
P05	47	M	T11	A	11	T	ISC	AM	Y	N	Y
P06	60	M	C6	D	15	T	ISC	None	Y	Y	Y
P07	47	M	C5	C	27	T	Sheath	AM	N	Y	Y
P09	56	M	T3	D	2	Non-T	Voids	AM	N	Y	Y

*Bladder management for storage (AM, Antimuscarinic medication) and voiding (ISC is Intermittent Self Catheterisation) is shown as is presence of self-reported bladder sensation and self-reported incontinence.*

### Stimulation Set-Up

**FIGURE 1:**
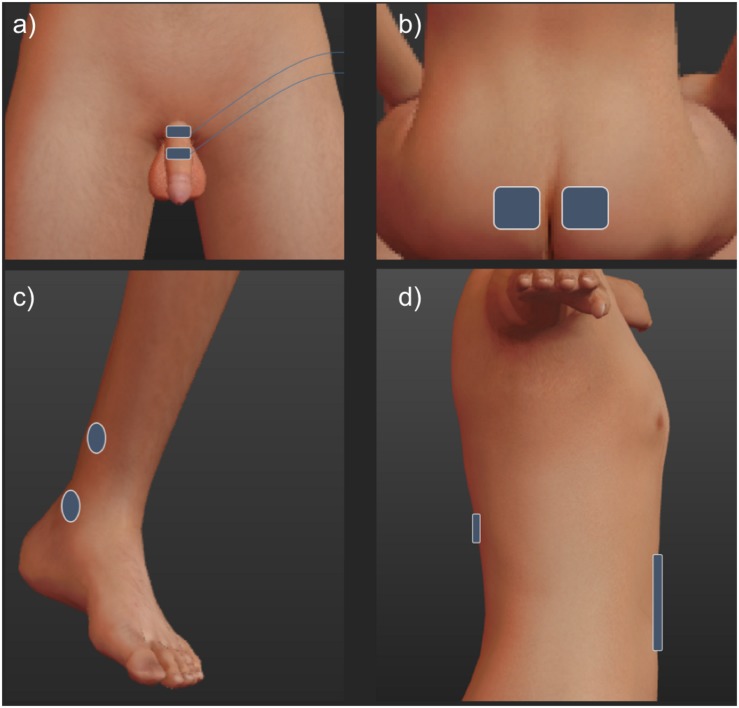
To stimulate the dorsal genital nerve **(a)**, electrodes were placed on the dorsum of the penile shaft. 1 cm paddle electrodes (Ambu Neuroline 710) were placed approximately 2 cm apart, the cathode was placed proximally. Tibial nerve electrodes **(c)** were 2.5 cm round surface electrodes (PALS) placed unilaterally 1 cm posterior and approximately 3 cm superior to the medial malleolus (cathode) and approximately 5 cm superior (anode). Sacral electrodes **(b)** were 5 × 5 cm (PALS) electrodes placed over either side of the sacrum, at the level determined to be over the S3 foramina through manual palpation of the sacrum. Spinal stimulation **(d)** used 5 cm circular and 7.5 × 10 cm electrodes (PALS) over T11-12 and the abdominal areas, respectively (Hofstoetter et al., 2014).

The stimulation sites are outlined in Figure 1. All stimulation pulses were monophasic, cathodic, 200 μS pulses delivered at 15 Hz from an electrically isolated constant current stimulator (DS7, Digitimer Ltd., United Kingdom).

To set the stimulation amplitude, 15 Hz bursts of 1 s were delivered at the relevant site, increasing the amplitude until either: twice the threshold for contraction of the external anal sphincter (EAS_thresh_), detected visually, was reached; a strong motor contraction was elicited in adjacent muscles; or stimulation was intolerable.

### Standard Cystometry

Standard cystometry (CMG) (Rosier et al., 2017) was performed at baseline and with stimulation. During all tests the participant was supine. A 10.5 Ch catheter was placed urethrally and used to fill the bladder with room temperature 0.9% saline at 60 ml/min. Pressure was measured using Medex (Smiths Medical Ltd.) pressure transducers placed at the level of the symphysis pubis, through 4.5 Ch water filled catheters placed urethrally to measure vesical pressure (P_*ves*_) and rectally to measure abdominal pressure (P_*abd*_). Detrusor pressure was calculated as P_*det*_ = P_*ves*_ − P_*abd*_. Infused volume was measured using a weight transducer. Signals were filtered and amplified using a CED 1902 isolated amplifier (2-pole Butterworth low pass, cut off 2000 Hz, gain 1200), digitised through a CED 1401 (sample rate 10 Hz) and recorded on Spike 2 software (Version 4, Cambridge Electronic Devices Ltd.) used to display data and trigger stimulation.

### Protocol

Each stimulation site was tested on separate days. During each session, a baseline control CMG was conducted, followed by up to three experimental CMGs where stimulation was applied conditionally (triggered manually) at a rise in detrusor pressure of 10 cmH_2_O, a further control CMG with no stimulation was then conducted. A gap of at least 5 min was left between each fill. The number of experimental CMGs was determined in agreement with participants on the day depending on time available. When possible, a further CMG using continuous stimulation throughout the fill was conducted following the second control CMG in TNS, SNS and SS sessions.

### Analysis

This protocol was designed to allow us to compare the effect of stimulation site within individuals vs. baseline taken on the same day, control fills were conducted before and after stimulation fills to try and evaluate any effect of repeat fills on cystometric capacity (Previnaire et al., 1996; Kirkham et al., 2001). Conditional stimulation triggered at a rise in detrusor pressure was used to determine the acute effects of stimulation on a detrusor contraction, whilst also allowing comparison of volume at first detrusor contraction during the same conditions in all fills (i.e., no stimulation).

**FIGURE 2:**
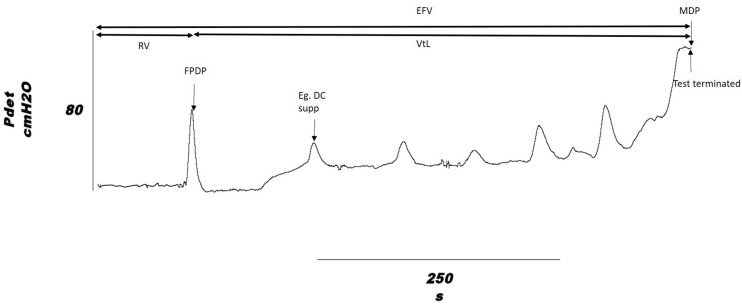
Outcome measures shown on a cystometry detrusor pressure trace, from a DGNS trial fill. Volume infused is taken as the outcome at the time point indicated on the pressure trace for Reflex Volume (RV) and End Fill Volume (EFV). VtL, Volume to Leakage; FPDP, First Peak Detrusor Pressure; MDP, Maximum Detrusor Pressure. An example of a suppressed detrusor contraction is highlighted.

The urodynamic outcomes measured were: volume infused at onset of first involuntary detrusor contraction, Reflex Volume (RV); bladder capacity, measured as the volume infused at end of fill, End Fill Volume (EFV), where end of fill is defined as when leakage occurred, participant was unable to tolerate sensation or when a detrusor contraction was sustained at > 45 cmH_2_O; Volume to Leakage (VtL), calculated as VtL = EFV − RV, which gives an indication of the direct effect of stimulation, removing the variation in RV that may cause EFV to vary; peak pressure during the first detrusor contraction, First Peak Detrusor Pressure (FPDP); Maximum Detrusor Pressure (MDP); and the number of suppressed detrusor contractions over the course of a fill. Each set of results had its own control recorded from the individual on the same day, change from baseline was used for comparison of stimulation sites. These are shown on a typical detrusor pressure trace in Figure 2.

The aim of this study was to quantify the effects of stimulation of four distinct sites on the ability of individuals with SCI and NDO to store urine at low pressure, at normal capacity and without incontinence. The assess this during CMG we quantified the change in bladder capacity, through the measures EFV and VtL, in bladder pressures, looking at FPDP, MDP and the number of suppressed detrusor contractions. We considered a detrusor contraction to be suppressed where it was not sustained, did not result in leakage or strong urge to void and peaked at a pressure below MDP. Average peak detrusor pressure was used as an additional measure where there was at least two detrusor contractions.

Stimulation of a site was considered successful at increasing capacity where EFV or VtL was increased by 100 ml or EFV was increased by 50%, and successful at decreasing storage pressures where FPDP was below 40 cmH_2_O and a detrusor contraction was suppressed.

All statistical analysis was conducted in Matlab (Version 2017b, Mathworks). A Wilcoxon signed rank test was used to look for significance of change from baseline in each site (matched pairs), with significance level set at *p* < 0.05. Data was tested using a Shapiro–Wilks test and subsequently compared using a Friedman’s test, as deemed suitable, to look for differences in the change in EFV between stimulation sites. A chi-squared test was conducted to look at the relationship between stimulation amplitude and change in VtL. All results, unless otherwise stated, are presented as mean ± standard deviation.

## Results

### Baseline Bladder Activity

End Fill Volume during control CMGs was 205 ± 109 ml and the mean change between pre and post stimulation control fills in a session was 13 ± 79 ml (*p* = 0.414). Change in EFV between pre and post stimulation control fills for individual sites was 23 ± 97 ml in DGNS tests, 36 ± 91 ml in TNS, 36 ± 55 ml in SNS and −48 ± 49 ml in SS.

Volume to Leakage in control fills was 42 ± 29 ml and change from pre- to post-stimulation control VtL was 6 ± 28 ml. RV across all fills (including stimulation fills, before stimulation was applied) was 167 ± 96 ml, the mean change from the first fill in each session was −1 ± 63 ml.

### Stimulation Amplitude

EAS contraction was elicited during stimulation threshold testing in 6/7 subjects during DGNS, 2/7 subjects during TNS, 5/7 during SNS, and 0/7 during SS. Stimulation amplitudes are shown in Table 2. 3/7 participants could tolerate DGNS at a level of 2xEAS_thresh_.

**TABLE 2:** Stimulation amplitude at sensory and motor thresholds and at maximum tolerable level.

	**DGNS**			**TNS**				**SNS**			**SS**		
				
**ID**	**Sensory**	**EAS**	**Max**	**Sensory**	**Toe**	**EAS**	**Max**	**Sensory**	**EAS**	**Max**	**Sensory**	**EAS**	**Max**
P01	NA	32	64	NA	20	45	60	NA	50	90	31	NA	40
P03	10	15	45	15	30	NA	30	7	50	70	15	NA	70
P04	2	NA	18	30	NA	NA	70	25	NA	70	–	–	–
P05	NA	37	74	NA	NA	NA	43	NA	55	80	–	–	–
P06	5	25	26	27	32	NA	75	7	50	70	5	NA	55
P07	12	30	40	15	24	55	75	10	43	90	10	NA	75
P09	3	20	20	3	NA	NA	30	10	NA	60	10	NA	55
Mean	6	27	41	18	27	50	55	12	50	76	14	NA	59
SD	4	7	20	10	5	5	19	7	4	10	9	NA	12
Med	5	27.5	40	15	27	50	60	10	50	70	10	NA	55

*NA indicates that no value was recorded (no sensation or no activity seen), – represents no tests were conducted. All results are in mA.*

### Effect of Stimulation on Urodynamic Outcomes

**FIGURE 3:**
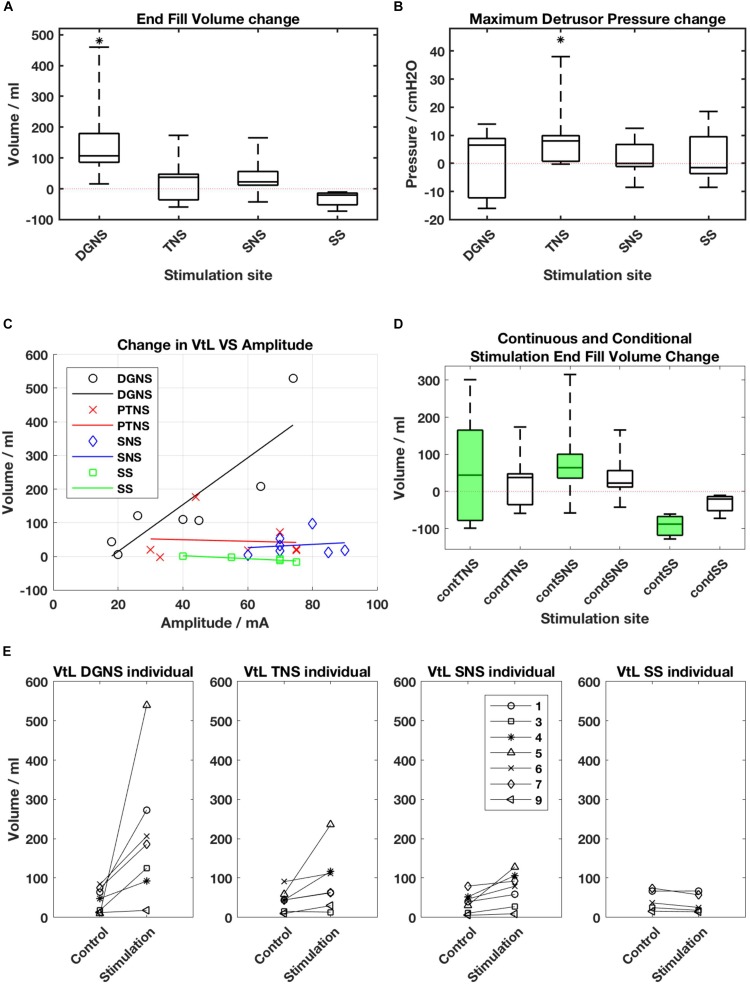
**(A)** Boxplot of end fill volume and **(B)** Maximum Detrusor Pressure change from baseline for each site. Boxes show median, interquartile ranges, and error bars denote the range. Stars denote that change from baseline is *p* < 0.05 following a Wilcoxon Rank Sum test. **(C)** Amplitude of stimulation vs. change in Volume to Leakage (VtL) for each site. DGNS appears to deliver increased gains when applied at greater amplitudes (*R*^2^ = 0.75). **(D)** Changes from baseline in end fill volumes (EFV) in continuous (green) and conditional (white) stimulation fills. Boxes represent the median and interquartile range of within session changes in EFV, whiskers show the range. **(E)** Individual changes in VtL as mean values obtained during control and conditional stimulation fills from each session.

All results are shown in Figure 3 and individual results for all urodynamic outcomes relating to volume are in Table 3 and relating to pressure are in Table 4. Dorsal Genital Nerve Stimulation increased EFV by 153 ± 146 ml (range 16–460 ml, *p* = 0.016), or 117 ± 201% (range 11–571%). VtL was increased by 161 ± 175 ml (range 6–530 ml, *p* = 0.016), or 950 ± 1784% (range 46–5300%). DGNS was able to suppress detrusor contractions in 5/7 participants, in whom the mean number of suppressed contractions was 2 ± 2 (range 1–6). At least one contraction was suppressed at a peak pressure of less than 40 cmH_2_O in all 5/7. Incontinence was prevented using DGNS in 3/6 participants who had leaked in control fills. FPDP was reduced by a mean of 34 ± 35 cmH_2_O (*p* = 0.078), or 42 ± 38%, to below 30 cmH_2_O in 3/7 participants. Average peak detrusor pressure, for just the five participants in whom at least one detrusor contraction was suppressed, was reduced by 22 ± 28 cmH_2_O (range −63 to +7, *p* = 0.156), or by 26 ± 31%. MDP was not changed by DGNS, 0 ± 12 cmH_2_O (*p* = 1.00).

**TABLE 3:** Bladder capacity results from baseline and conditional stimulation fills.

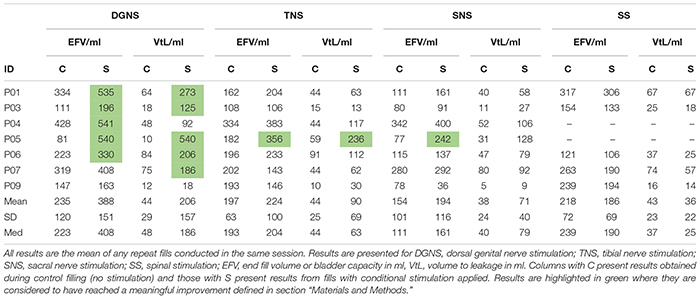

**TABLE 4:** Detrusor pressure results from baseline and conditional stimulation fills.

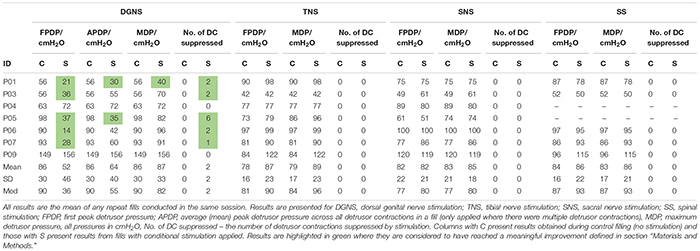

Tibial Nerve Stimulation did not visibly suppress a detrusor contraction in any participant, consequently FPDP was the same as MDP as all first detrusor contractions were terminal for the test. MDP was increased by 10 ± 13 cmH_2_O (range 0–38 cmH_2_O, *p* = 0.031), or 11 ± 16%. Some changes in bladder capacity were seen, however. Increases in EFV were inconsistent (*p* = 0.680) but VtL was increased by 46 ± 62 ml (range −2 to 177 ml, *p* = 0.031), or 110 ± 108% (range −13 to 299%).

Sacral Nerve Stimulation similarly did not clearly suppress any detrusor contractions and changes in EFV and MDP were not significant (*p* = 0.680 and *p* = 0.625). VtL was increased by 34 ± 33 ml (range 4–98 ml, *p* = 0.016), or 113 ± 94% (range 16–320%).

Spinal Stimulation did not significantly change outcomes, though trends were observed in the smaller (*n* = 5) cohort. EFV decreased in all (*n* = 5) participants by −33 ± 26 ml (range −11 to −73 ml, *p* = 0.063) and VtL by −7 ± 7 ml (range −12 to −1 ml, *p* = 0.125), or −18 ± 12% (range −32 to 1%). MDP was unchanged (*p* = 1.00).

A Friedman’s test was used to compare the EFV changes across the four sites. The first test included all seven participants, but compared only between the DGNS, TNS and SNS sessions, as 2/7 were unable to trial SS. The *p*-value was 0.0038 and *post hoc* analysis found the DGNS session’s change in EFV to significantly differ from both SNS and TNS. A second test was conducted across all four sites, with results from 5/7 included. *P* = 0.0036, and *post hoc* analysis showed only DGNS and SS results to differ significantly from one another.

### Comparison of Continuous and Conditional Stimulation

Continuous stimulation mode was applied using TNS in *n* = 5, SNS in *n* = 5 and SS in *n* = 3. We found no significant change from baseline in either RV or EFV. TNS produced a change of 59 ± 161 ml (*p* = 0.625) in EFV and 6 ± 83 ml (*p* = 1.00) in RV; SNS a change of 87 ± 125 ml (*p* = 0.156) in EFV and 44 ± 58 ml (*p* = 0.156) in RV; and SS, a change of −92 ± 34 ml (*p* = 0.25) in EFV and −85 ± 42 ml (*p* = 0.25) in RV.

## Discussion

This investigation was a pilot study to compare the acute urodynamic effect of transcutaneous stimulation at four anatomical sites. In the participants tested, DGNS was the most effective at increasing bladder capacity and reducing leakage. Only DGNS had a meaningful effect on bladder capacity and detrusor pressure, demonstrated by increasing volumes by at least 100 ml or 50% and decreasing peak detrusor pressure in 5/7 participants. Though DGNS, TNS and SNS all lead to increases in volume from RV to EFV, VtL. Only TNS lead to any consistent change in MDP, increasing it by 10 ± 13 cmH_2_O (*p* = 0.0313), which may be a worrying effect of neuromodulation attempts requiring further investigation, this is discussed further in section “TNS Effects.”

### Stimulation Amplitude

The maximum tolerable amplitude was used in all sites, apart from in participants with no sensation where either twice the EAS_thresh_ or an amplitude that did not evoke strong contractions in adjacent muscles was used. During set up, twice the EAS_thresh_ was used as a target based upon previous research of DGNS (Previnaire et al., 1996). In animal models of TNS and SNS, 3–4 times the motor threshold for toe twitching is required to suppress detrusor activity (Su et al., 2012; Kovacevic and Yoo, 2015). This was used as an amplitude targets for TNS and SNS, but in practice we were not always able to reach these targets. The average DGNS amplitude was 41 mA, 1.7xEAS_thresh_, and TNS was 55 mA, 2.6xToe_thresh_. SNS had no observable effect on the toes, though both TNS and SNS elicited a visible contraction in the EAS in 2/7 and 5/7 participants, respectively. SNS, applied using magnetic stimulation but not transcutaneous stimulation, has been shown to stimulate pudendal efferent fibres, generating EAS responses at short latencies (Eardley et al., 1990; Sheriff et al., 1996). Two studies provide evidence of a Tibial-Anal reflex pathway in the literature, showing long latency responses of over 90 ms (Mai and Pedersen, 1976; Pedersen et al., 1978). Neurophysiological study of the link between neuromodulaton site and EAS or urethral sphincter activity may provide useful information regarding both the mechanism of neuromodulation techniques and optimal electrode placement. Our study of SS as an intervention to suppress involuntary detrusor contractions did not use electromyography of either sphincter or lower limb activity during set up. During SS set up we were unable to visually detect any EAS response and only reached amplitude to evoke a visually detectable lower limb response in P07, subsequently stimulating just below this threshold. In other participants strong contraction of adjacent musculature possibly prevented us reaching sufficient amplitude to elicit a detectable lower limb response. It is possible that target structures were not stimulated using the parameters described including electrode position and this uncertainty is a weakness in our study. The lack of electrophysiological data in set up of SS is a limitation of this study that should be remedied in future acute study of SS for bladder control. For future investigation of neuromodulation sites, we recommend thorough assessment of this during stimulation set up.

### Changes in Post Reflex Volume Capacity

The volume infused between the onset of the first detrusor contraction and the end of the fill (VtL) gives an indication of the direct effect of stimulation, removing the variation in RV that may cause EFV to vary. This measure may also give us an indication of the acute changes that stimulation produces. As we filled the bladder continuously at 60 ml/min this also represents the time (s) from RV to leakage or intolerable urgency in these non-physiological conditions. At baseline this was 42 ± 29 ml. DGNS increased VtL to 206 ± 157 ml, TNS to 90 ± 69 ml, SNS to 71 ± 40 ml and SS decreased VtL to 36 ± 22 ml. This demonstrates the capacity of DGNS for use in a conditional neuromodulation system for people with SCI, however, also suggests that TNS and SNS are suitable for further evaluation.

### TNS Effects

Transcutaneous TNS has increased bladder capacity in subjects with SCI when applied at 10 Hz and just below the amplitude required to elicit a toe twitch (Amarenco et al., 2003); and 25 Hz and 30 mA (Andrews and Reynard, 2003). In both of these studies continuous stimulation was used, it was reported to suppress detrusor contractions and increase capacity by between 75 and 170 ml.

**FIGURE 4:**
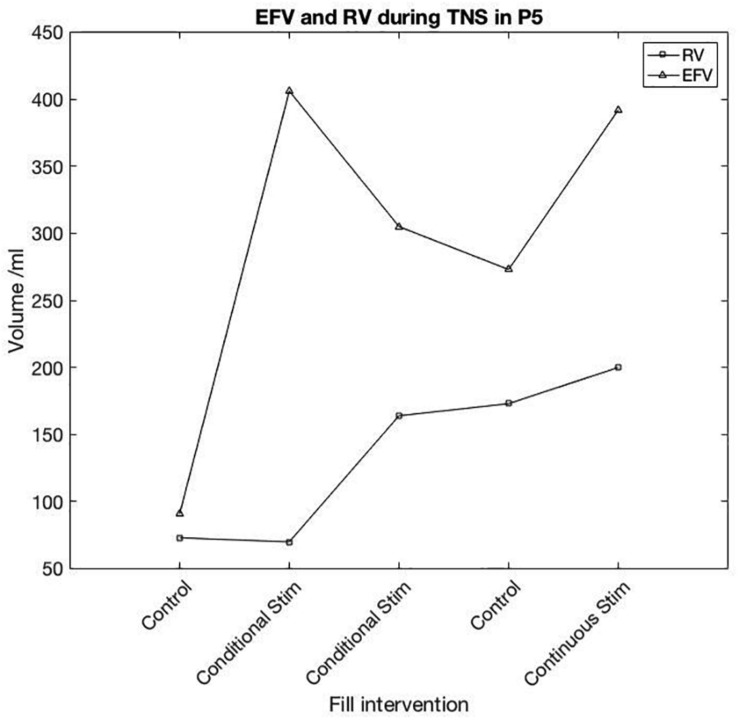
Participant 5 TNS session results. First reflex detrusor contraction (RV) and End Fill Volume (EFV) are shown for each fill in the session. Following the first conditional TNS fill, a noticeable change in volume prior to the first detrusor contraction was seen in each subsequent fill both with and without stimulation.

One participant appeared to respond in a similar fashion to previous positive reports. In this study, P05 showed a large increase in capacity in response to TNS, whilst the detrusor contraction was not clearly suppressed, instead appearing as compound NDO without leakage. Bladder capacity was increased from 91 ml in pre-stimulation control, to 406 and 303 ml with conditional stimulation. Following conditional TNS, control EFV was 273 ml and with continuous stimulation EFV was 372 ml. For pre-stimulation control (baseline) and conditional-stimulation fill 1 (i.e., until stimulation was first applied) RV was 73 and 70 ml. Following the first stimulation fill, RV was 164, 173, and 200 ml in conditional stimulation, control and continuous fills, respectively. P05’s individual fill results are shown in Figure 4.

In each of the other sessions, where P05 trialled different sites, post-stimulation control was similar to pre-stimulation control, suggesting TNS may have some carry-over effect on bladder capacity where other sites did not. P05’s response to DGNS and SNS was the most positive in this participant group, mean EFV was increased by 459 ml during DGNS and 165 ml during SNS.

P05’s TNS response is similarly positive to those reported in the literature, but is alone in the seven participants tested here (Amarenco et al., 2003; Andrews and Reynard, 2003; Kabay et al., 2008), emphasising the need to investigate whether this is repeatable, how to best elicit a response, or predict a responder, and the need for a larger trial. An important consideration is that whilst TNS did increase capacity, in the same person DGNS improved all parameters to a far greater extent as evident in individual results presented in Figure 3E and Table 3.

Whilst bladder capacity was markedly increased in one participant, our analysis of the seven participants studied, found no significant effect of TNS on bladder capacity. However, we did find a significant increase in both VtL and MDP. We also saw a response in the anal sphincter in 2/7 participants. It may be that TNS acts on the EAS and the urethral sphincter but is unable to suppress the detrusor simultaneously as DGNS does, perhaps suggesting that small increases in capacity are allowed by increased sphincter activity whilst detrusor contractions remain unsuppressed. The potential for increased MDP represents a real risk to the upper urinary tract and should be carefully measured in future work.

### SNS Effects

Sacral Nerve Stimulation has a direct suppressive effect on provoked detrusor contractions when magnetic stimulation is applied at an amplitude above that required to evoke a big toe contraction (Sheriff et al., 1996) and when mixed nerves of S2–4 are stimulated using an implanted Finetech-Brindley stimulator (Kirkham et al., 2002). By applying surface electrodes over the sacrum and applying maximum tolerable stimulation at 15 Hz, we found SNS had no observable effect on NDO in each of the seven patients tested, with no significant effect on MDP or EFV. VtL, however, was significantly increased (*p* = 0.016) suggesting stimulation does produce some effect on promoting continence either through sphincter activation, detrusor inhibition or a combination. A similar urodynamic study using surface electrodes, with MS participants, also found no effect on NDO (Fjorback et al., 2007a). We saw similar activation of the anal sphincter using transcutaneous stimulation to magnetic stimulation, suggesting recruitment of similar structures. However, it seems likely, based on successful reports, that it requires more selective, deeper penetrating, stimulation to elicit the effect reported using magnetic stimulation, and that transcutaneous stimulation in this position is unable to provide this. The reported effect of continuous sub- motor threshold stimulation was not tested here and may be worth pursuing in a chronic setting following reports from other groups, though not in people with SCI (Barroso et al., 2013; Quintiliano et al., 2015).

### SS Effects

Spinal Stimulation, applied at 15 Hz, reduced bladder capacity by 33 ± 26 ml when applied conditionally and by 85 ± 42 ml in the three participants who trialled continuous stimulation. The array of stimulation parameters reported in the literature is wide, here we tested SS with the parameters set to the same as the other sites, based on previous work in our centre finding 15 Hz stimulation to be appropriate for storage (Kirkham et al., 2002). The decrease in bladder capacity seen here may be due to increased excitability of spinal reflex activity, the result of increased abdominal pressure caused by contraction of abdominal muscles or given the sample size, within the natural range of change seen in repeat CMGs. Changes in EFV were not found to be significant (*p* = 0.06).

Urodynamic assessment of the acute effect of SS on NDO has recently been found to facilitate voiding (Gad et al., 2018; Herrity et al., 2018; Niu et al., 2018) at 1 Hz and improve storage dysfunction at 30 Hz (Gad et al., 2018). These improvements were reported to be frequency and location dependant, also applying high frequency carrier within stimulation pulse which may be why use of much higher amplitudes was possible. When applied continuously, at 15 Hz and 59 ± 12 mA, we found SS to decrease RV (from control) by 85 ± 42 ml, while Gad et al. found an increase in RV of 80 ± 50 ml in seven participants, during continuous 30 Hz stimulation. In the presented work we have trialled only one electrode configuration and frequency (15 Hz) and have not assessed voiding efficiency. Further acute study of SS at varying frequencies during storage and micturition would be valuable, as would the comparison of epidural and transcutaneous approaches.

### DGNS Effects

Dorsal Genital Nerve Stimulation has been repeatedly shown to acutely inhibit NDO in supra-sacral SCI. A recent meta-analysis of eight acute studies, with a total of 97 SCI subjects, showed a change in bladder capacity from baseline of 131 ± 101 ml (Bourbeau et al., 2018a). The increase in EFV of 153 ± 146 ml we report here is comparable to past studies of DGNS, as is the number of successive suppressed contractions (1–6). The meta-analysis showed a link between DGNS amplitude and bladder capacity gains, a trend also found in our results, Figure 3C.

In this study we found 5/7 participants responded to conditional DGNS with increased bladder volumes (EFV or VtL) of at least 100 ml or 50% and with at least one detrusor contraction being suppressed at a peak pressure of below 40 cmH_2_O. Two participants (P01 and P05) responded with large increases in bladder capacity to over 500 ml and decreases in average peak detrusor to under 40 cmH_2_O, eliminating incontinence. These two participants both had ASIA A SCI with no bladder or genital sensation, allowing use of 2xEAS_thresh_. The other three responders (P03, P06, and P07) all showed increased bladder volumes and at least one suppressed detrusor contraction peaking at below 40 cmH_2_O, however, average and maximum peak detrusor pressures rose above this threshold. These three participants all had some retained bladder and genital sensation. It is of interest that for those with sensation, DGNS may provide a way of decreasing peak pressures during initial detrusor contractions whilst appropriate toileting arrangements are made, thus preserving continence and low pressure storage without MDP ever being reached. Whilst those (such as P01 and P05) without sensation, for whom some form of feedback on bladder activity would be required to initiate preparation for voiding, may use DGNS to prolong continence, increase bladder capacity and decrease storage pressures when applied at a higher amplitude.

The amplitude of DGNS required to optimally suppress detrusor contractions is twice EAS_thresh_ (Previnaire et al., 1996; Kirkham et al., 2001; Brose et al., 2018). Here, 3/7 participants were able to tolerate this amplitude, all responding well to DGNS, two of whom had complete lesions with no pelvic sensation. Other participants trialled stimulation at 1×, 1.04×, and 1.33×EAS_thresh_ and in the remaining one participant we found no EAS response at the 18 mA amplitude they found tolerable. A study of 23 incomplete SCI subjects found DGNS to be tolerable and effective (Brose et al., 2018), showing DGNS may be applicable across a broad range of SCI patients. DGNS has been applied with success as a home based intervention in short pilot studies, reducing incontinence episodes and increasing voided volumes (Lee and Creasey, 2002; Lee et al., 2012; Bourbeau et al., 2018b). This success is tempered by problems found with chronic use of available surface electrodes, particularly with female users, and the lack of an effective trigger to “close the loop” in a conditional neuromodulation system for those with no pelvic sensation (Martens et al., 2010; Lee et al., 2012). The location of electrodes on the penis or clitoris may be unacceptable to some patients, which makes evaluating alternatives important.

### Study Limitations

Our results provide insight into the practical application, and within individual comparison, of DGNS, TNS, SNS, and SS. However, they must be interpreted within the context of a small sample size, fixed stimulation parameters and limited repeats for each subject.

## Conclusion

Within this pilot study, we present beneficial effects of DGNS in suppressing detrusor activity and increasing bladder capacity. DGNS, TNS, and SNS all increased the volume held following initial detrusor contraction. TNS also lead to a increase in MDP. SS was trialled in five people, in whom small decreases in bladder capacity were observed.

The small changes observed in TNS, SNS, and SS require further exploration and their potential should not be discounted. Examining their interactions with the neural control of LUT in an acute setting, alongside study of stimulation parameters, could inform future protocols. DGNS has a clear and robust effect on NDO and practical challenges in its chronic use and applicability should be addressed.

In conclusion, NDO following SCI continues to present an important clinical problem with limited solutions available for chronic management. Transcutaneous stimulation is an interesting and non-invasive potential treatment option, requiring further research to understand its effect and range of applications. This study is the first direct comparison of the effect of transcutaneous stimulation sites on NDO in SCI participants. We found that DGNS was the only effective site for improving storage of urine in the group of people with SCI tested.

## Data Availability Statement

All datasets generated for this study are included in the article/supplementary material.

## Ethics Statement

The studies involving human participants were reviewed and approved by Queen Square Regional Ethics Committee, NHS Health Research Authority. The patients/participants provided their written informed consent to participate in this study.

## Author Contributions

SD designed the study, performed the experiments, analysed the data, wrote the initial manuscript, and edited the final manuscript. AV designed the study and edited the final manuscript. LD and RH edited the final manuscript. SK designed the study, performed the experiments, and edited the final manuscript.

## Conflict of Interest

The authors declare that the research was conducted in the absence of any commercial or financial relationships that could be construed as a potential conflict of interest.
